# Characterising microstructural retinal changes in children with inherited retinal dystrophies – a retrospective observational cross-sectional study

**DOI:** 10.1007/s00417-025-06983-7

**Published:** 2025-10-15

**Authors:** Jonathon Holland, Brent Rodda, Jonathan B. Ruddle, Marianne EF Piano, Elisse J. Higginbotham, Lauren N. Ayton, Jasleen K. Jolly

**Affiliations:** 1 Department of Clinical Neurosciences, University of Cambridge, Bay 13, Clifford Albutt Building, Cambridge Biomedical Campus, Cambridge, CB2 0AH UK; 2 Department of Optometry and Vision Sciences, The University of Melbourne, Melbourne, Australia; 3 Department of Ophthalmology, Royal Children’s Hospital, Melbourne, VIC Australia; 4 Centre for Eye Research Australia, Royal Victorian Eye and Ear Hospital, East Melbourne, Australia; 5 National Vision Research Institute, Australian College of Optometry, Carlton, Australia; 6 Department of Surgery (Ophthalmology), The University of Melbourne, Melbourne, Australia; 7 Jolly Vision Science, Cambridge, UK

**Keywords:** Optical coherence tomography, Retina, Eye diseases, hereditary, Retinal dystrophies, Child, Humans

## Abstract

**Purpose:**

Trials exploring novel treatments for inherited retinal diseases (IRDs) have utilised microstructural retinal changes for outcome measures, but such changes in children are incompletely described. We aimed to characterise the retinal microstructural in children with IRDs versus controls using optical coherence tomography (OCT).

**Methods:**

Cross-sectional study of retrospective OCT data from 51 children (91 eyes) with IRDs attending the Melbourne Children’s Eye Clinic and 64 controls (126 eyes) recruited prospectively at Australian College of Optometry clinics.

OCT was performed using a Nidek RS-3000 with volume scans in each participant eye. Image quality was manually reviewed. Semi-automated layer segmentation was performed and thickness based on eccentricity relative to the central fovea position was calculated to standardise measurements between participants.

**Results:**

Inner retinal layer thickness was significantly increased in IRD groups versus controls (rod-cone *p* < 0.0001; cone-rod *p* < 0.0001; macula *p* < 0.0001; cone *p* < 0.0001). This was not observed for photoreceptor complex thickness where instead thinning versus controls was seen in cone-rod disorders (rod-cone *p* = 0.89; cone-rod *p* < 0.0001; macula *p* = 0.21; cone *p* = 0.17).

Total thickness was significantly reduced in cone and cone-rod group versus controls, but not in other IRD types (rod-cone *p* = 0.25; cone-rod *p* < 0.0001; macula *p* = 0.74; cones *p* = 0.004).

**Conclusion:**

Our findings suggest retinal remodelling takes place in childhood-onset IRDs. Clinical trials in IRDs should consider remodelling when evaluating treatment effects. Future studies on the longitudinal natural history of retinal microstructural changes in childhood IRDs are required to address the impact of factors including IRD type, sex, age and disease duration.

**Supplementary Information:**

The online version contains supplementary material available at 10.1007/s00417-025-06983-7.

## Key messages

*****What is known***:**

- Inherited retinal disease (IRD) trials have employed optical coherence tomography (OCT) based outcomes; however, microstructural OCT changes in childhood IRDs are less studied.

*****What is new***:**

- We found the retinal microstructure of children with IRDs differed from controls, with different IRD types demonstrating different patterns of change.
- This may indicate potential early retinal remodelling in these conditions.
- Our findings highlight a need for greater understanding of retinal microstructural changes in childhood IRDs, to successfully develop OCT-based clinical trial outcome measures.

**Supplementary Information:**

The online version contains supplementary material available at 10.1007/s00417-025-06983-7.

## Introduction

Inherited retinal diseases (IRDs) consist of a large group of individually rare conditions with a wide range of clinical presentations and underlying causes, including more than 340 specific genetic loci [1]. Altogether, they constitute the leading cause of legal blindness amongst working-age populations, and the second leading cause in children, in both Australia [2] and the UK [3]. With the advent of potential targeted gene therapies for many IRDs [4], it is essential to understand the natural history of early microstructural retinal changes in these conditions, because these may represent potential clinical trial outcome measures [5].

Furthermore, there is limited and conflicting evidence regarding post-treatment microstructural changes in such clinical trials – for example in patients treated with voretigene neparovovec, a gene therapy for *RPE65*-related IRD delivered by sub-retinal injection, both subsequent retinal regeneration and thinning have been observed [6]. A better understanding of the baseline changes in a paediatric population, rather than extrapolation from adult natural history work, may help to contextualise findings seen, particularly as future gene therapy trials expand to paediatric populations.

Optical coherence tomography (OCT), a light interferometry technique, enables visualisation and objective measurement of retinal structural changes in IRDs, with high resolution and fast acquisition times [7]. However, there is a paucity of data describing OCT changes in IRDs amongst children [5]. We aimed to characterise a range of IRDs in children through a cross-sectional multi-centre study using OCT, through comparison to age-matched children without IRDs. We analyse the retinal thickness and key features seen in different IRDs in a paediatric population presenting to ophthalmology and optometry centres in Melbourne, Australia.

## Methods

OCT data from all children with a clinical diagnosis of an IRD attending the Melbourne Children’s Eye Clinic (a specialist paediatric ophthalmology practice) between April and August 2021 were collected retrospectively.

The patient populations were separated into four groups, based upon which photoreceptor class is affected first and most severely – as determined by the phenotype findings and predicted function of the molecular genetic diagnosis (where known). These groups were rod-cone dystrophies, cone-rod dystrophies, cone dystrophies, and macular dystrophies.

Prospectively, we collected age and sex-matched control data using the same OCT scan specifications in a cohort of healthy paediatric volunteers at the Australian College of Optometry, with data collection by clinicians EH and MP between July 2021 and September 2022.

OCT was performed using RS-3000 Advance (Nidek, Japan) and volume scans attempted for each participant in each eye (Nidek macular map, disc map and horizontal raster macula line scan [9 × 9 mm 512 A-scans x 128 B-scans, or 256 A-scans in younger children]).

Image quality was reviewed visually by a Paediatric Ophthalmologist with expertise in IRD (JR) but low quality data was accepted if layers were visible, due to the inherent challenges with imaging children [8].

Segmentation of the layers was performed using a semi-automated method implemented in ImageJ using Trainable Weka Segmentation. Small areas of the retina were manually allocated to their respective layers. A pixel-by-pixel segmentation of the retina was then automatically generated based on the colour and intensity of the manually selected areas. Layer definitions based on were based on the APOSTEL [9] definitions and the In.OCT consensus [10].

**Fig. 1:**
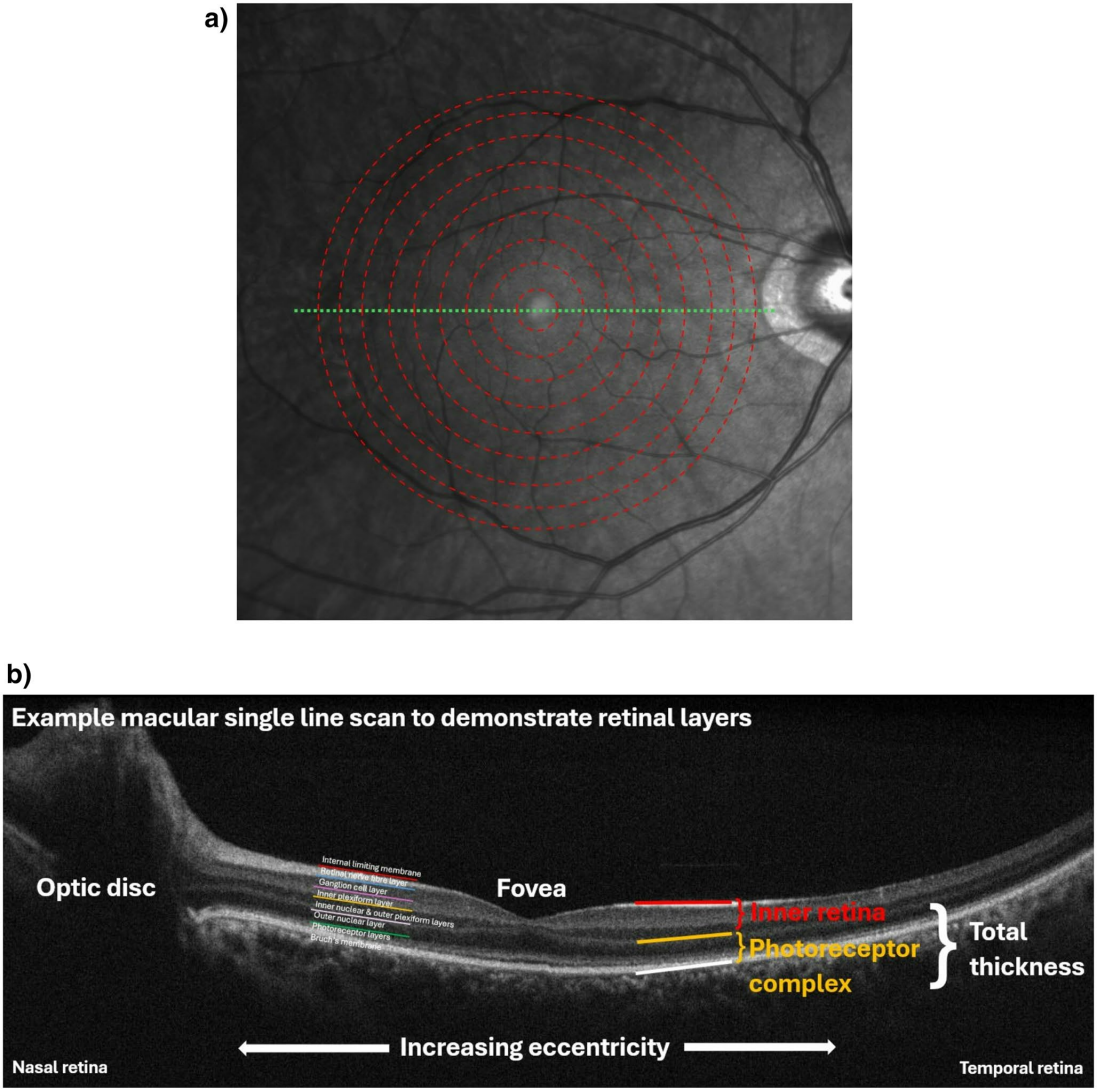
**(a)** Illustration of derivation of macular eccentricity relative to the fovea - dashed/red lines – taken from multiple raster scans across the retina – the dotted/green line; single example shown. **(b)** Annotated single macular line scan (indicated by dotted/green line in a) demonstrating appearances of different retina layers and grouping applied for subsequent analyses. **Descriptive caption: (a)** an image showing a human retina as seen using infrared imaging. A series of concentric dashed lines form rings centred on the fovea, with a dotted line perpendicular to the concentric rings in the horizontal plane transecting the fovea. **(b)** a diagram showing a section of human retina acquired using OCT. Annotations highlight the relative positions of the different retinal layers, how eccentricity relates to the fovea, and the larger grouped layer categories used in our analysis

The thickness of the inner retina (IR) was defined by the internal limiting membrane to edge of the outer plexiform layer. The photoreceptor complex (PRC) was defined as the sum of the outer nuclear layer, and inner and outer segment thickness as measured to the border of the RPE/Bruch’s membrane. OCT measurements were standardised between all participants by calculating thickness based on eccentricity relative to the central fovea position (Fig. 1), using previously published methodology [11]. These standardised measurements represent the outcome measure for the study.

This work adhered to the Declaration of Helsinki, and all participants provided informed consent. Ethics approval was granted through the University of Melbourne Human Research Ethics Committee (#21179 for retrospective IRD data, and #21663 for the prospective control data).

Statistical analyses were performed using R version 4.3.2 (R Core Team, 2023), RStudio (Posit Team, 2024) with the following packages: Tidyverse (Wickham et al., 2019); Patchwork (Pedersen T, 2024); ggpubr (Kassambara A, 2023), dunn.test (Dinno A, 2024), gridExtra (Auguie B, 2017), mgcv (Wood S, 2023), and gratia (Simpson G, 2024).

Each dataset was examined for normality through informal assessment of histograms, QQ-plots and using the Shapiro-Wilk test; all data except for cone group IR, macula group PRC left eye and rod-cone group IR left eye was non-normally distributed and non-parametric tests were subsequently employed. For each group there was strong left-right correlation between eyes and a mean value of both eyes in each patient was taken to simplify further analyses and reduce the impact of missing eye data.

To test for statistical difference between each IRD group and relevant age-matched control group (+/- 1 year), Kruskal-Wallis tests were performed across all degrees of eccentricity (test statistic reported as H(*degrees of freedom)* = and the associated level of significance) and were followed by post-hoc Dunn tests with Bonferroni correction for multiple comparisons, to more precisely identify at which eccentricities any observed differences were seen.

The STROBE cross-sectional reporting guidelines were observed in preparation of the manuscript [12].

## Results

 Table 1 outlines baseline demographic characteristics of participants; Table 2 lists diagnoses amongst IRD participants. 8 participants had IRDs associated with systemic disorders: gyrate atrophy (n=1), Usher syndrome (n=1), neuronal ceroid lipofuscinosis (n=3), Bardet-Biedl syndrome (n=3); the 2 participants had diagnoses of Leber’s congenital amaurosis with *SPATA7*variants, which are not associated with systemic difficulties. Overall, 30 participants had relevant genetic variants associated with IRD identified (including those associated with the systemic disorders above); the remaining participants either had not undertaken genetic testing, had no results available, or had no known variants associated with retinal disease found. Thickness data in different layers for each group is summarised in supplementary information online (online resource 1).

**Table 1:** Participant demographics and visual acuity. **Descriptive caption: **a table comparing the mean age, self-reported biological sex, and mean visual acuity, for healthy volunteers, participants with rod-cone, cone-rod, macula and cone IRD groupings

	Healthy volunteers (*n* = 64)	Rod-cone IRD (*n* = 27)	Cone-rod IRD (*n* = 11)	Macula IRD (*n* = 9)	Cone IRD (*n* = 4)
**Mean age (SD; range)/years**	11.5 (3.1; 6–18)	11.7 (4,3; 4–18)	12.5 (3.8; 5–17)	10.0 (3.8; 4–16)	11.9 (1.7; 10–14)
**Male: Female**	36:28	18:9	7:4	5:4	2:2
**Mean visual acuity LogMAR (range)**	**0.00 (−0.20-0.40)**	**0.36 (0.00–1.30.00.30)**	**0.40 (0.05–1.10)**	**0.40 (0.05–1.00.05.00)**	**0.88 (0.70–1.10)**

**Table 2:** IRD diagnoses (clinical and/or genetic) amongst participants. **Descriptive caption: **a table with 3 columns and 28 rows. The first column indicates one of 27 different IRD diagnoses; the second column indicates the grouping IRD type we have applied; the third column indicates the number of participants in that diagnosis group

Diagnosis	Group	*n*
Achromatopsia	Cone	2
Best disease	Macula	3
Blue cone monochromatism	Cone	2
Congenital stationary night blindness	Rod-cone	2
Choroideremia	Rod-cone	2
CLN2-related macula dystrophy	Macula	1
CLN3-related cone-rod dystrophy	Cone-rod	2
Cone-rod dystrophy (*PROM1*)	Cone-rod	1
Cone-rod dystrophy (Other)	Cone-rod	3
Cone dystrophy (autosomal dominant)	Cone-rod	2
Cone dystrophy (other)	Cone-rod	3
Gyrate atrophy of retina	Rod-cone	1
Leber’s congenital amaurosis	Rod-cone	2
Macula dystrophy	Macula	2
Retinal dystrophy (*KIF11*)	Rod-cone	1
Retinal dystrophy (Usher syndrome)	Rod-cone	1
Retinal dystrophy (other)	Rod-cone	4
Retinitis pigmentosa (*IFT140*)	Rod-cone	1
Retinitis pigmentosa associated with Bardet-Biedl syndrome	Rod-cone	3
Retinitis pigmentosa (dominant)	Rod-cone	1
Retinitis pigmentosa (*CRB1*)	Rod-cone	1
Retinitis pigmentosa (*NR2E3*)	Rod-cone	1
Retinitis pigmentosa (other)	Rod-cone	1
Rod-cone dystrophy (other)	Rod-cone	1
Stargardt disease (*ABCA4*)	Macula	1
X-linked retinitis pigmentosa (*RPGR*)	Rod-cone	2
X-linked retinitis pigmentosa (*RP2*)	Rod-cone	1
X-linked retinitis (other)	Rod-cone	2

### Inner retinal layers

**Fig. 2:**
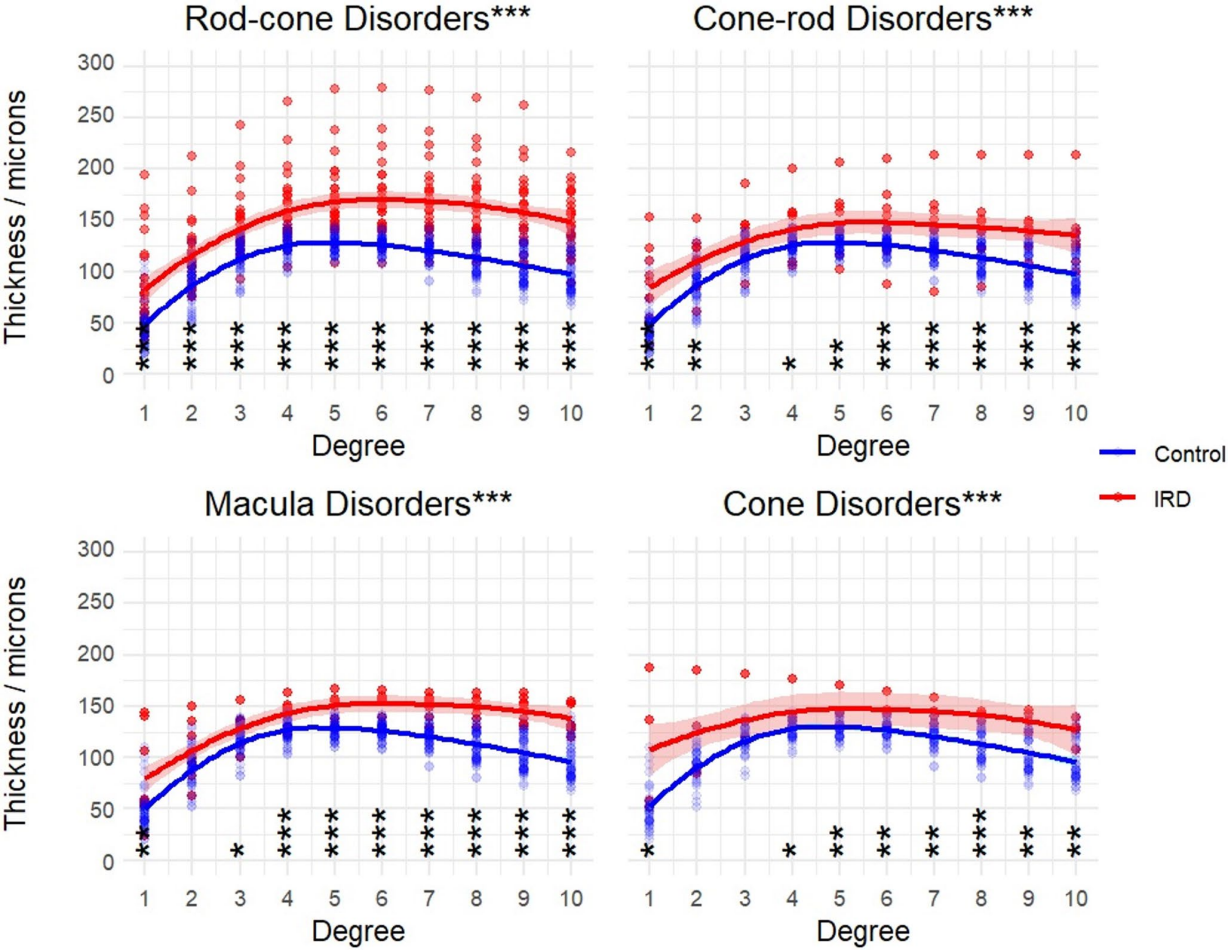
Inner retinal layer thickness was significantly increased in all IRD groups versus age-matched controls. Shading indicates standard error. P-values for post-hoc Dunn tests with Bonferroni correction displayed for each degree (* <0.025, **<0.01, ***<0.001). **Descriptive caption**: 4 x-y scatter plots comparing inner retinal thickness in microns on the y axis and degree on the x axis, for each IRD group. In each plot there is an upper line with error shading showing the increased thickness in the IRD groups, versus the lower line without error shading, showing the results from the control group

All IRD groups demonstrated significant thickening of the inner retinal layers versus within group age-matched controls across the measured area (Fig. 2. Kruskal-Wallis H test: rod-cone H(1) = 205.0, *p* < 0.0001; cone-rod: H(1) = 58.1, *p* < 0.0001; macula: H(1) = 87.2, *p* < 0.0001; *p* < 0.0001; cone: H(1) = 44.6, *p* < 0.0001).

### Photoreceptor complex

**Fig. 3:**
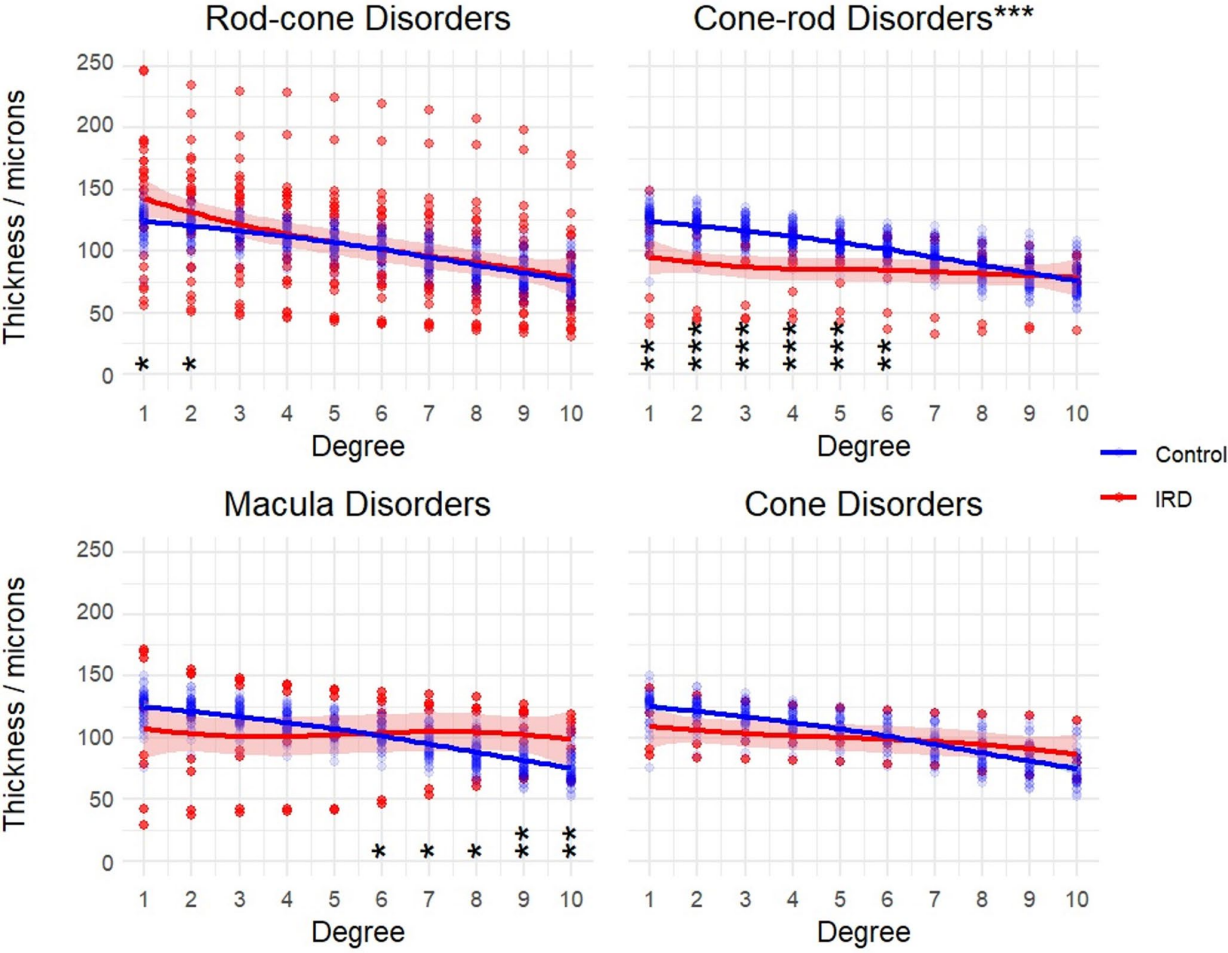
Photoreceptor complex layer thickness was significantly thinner in the cone-rod IRD group overall and specifically in degrees 1–6, but no significant difference overall was seen between other IRD groups and age-matched controls. Shading indicates standard error. P-values for post-hoc Dunn tests with Bonferroni correction displayed for each degree (* <0.025, **<0.01, ***<0.001). **Descriptive caption**: 4 x-y scatter plots comparing photoreceptor complex thickness in microns on the y axis and degree on the x axis, for each IRD group. In each plot the line with error shading indicates the IRD groups, versus the line without error shading, showing the results from the control group

There were no overall significant differences between photoreceptor complex thicknesses in IRD and age-matched control groups except for cone-rod disorders, where thinning was observed particularly in the macula (Fig. 3. Kruskal-Wallis H test: rod-cone H(1) = 0.02, *p* = 0.89; cone-rod H(1) = 40.3, *p* < 0.0001; macula H(1) = 1.6, *p* = 0.21; cone H(1) = 1.8, *p* = 0.17.

### Total thickness

**Fig. 4:**
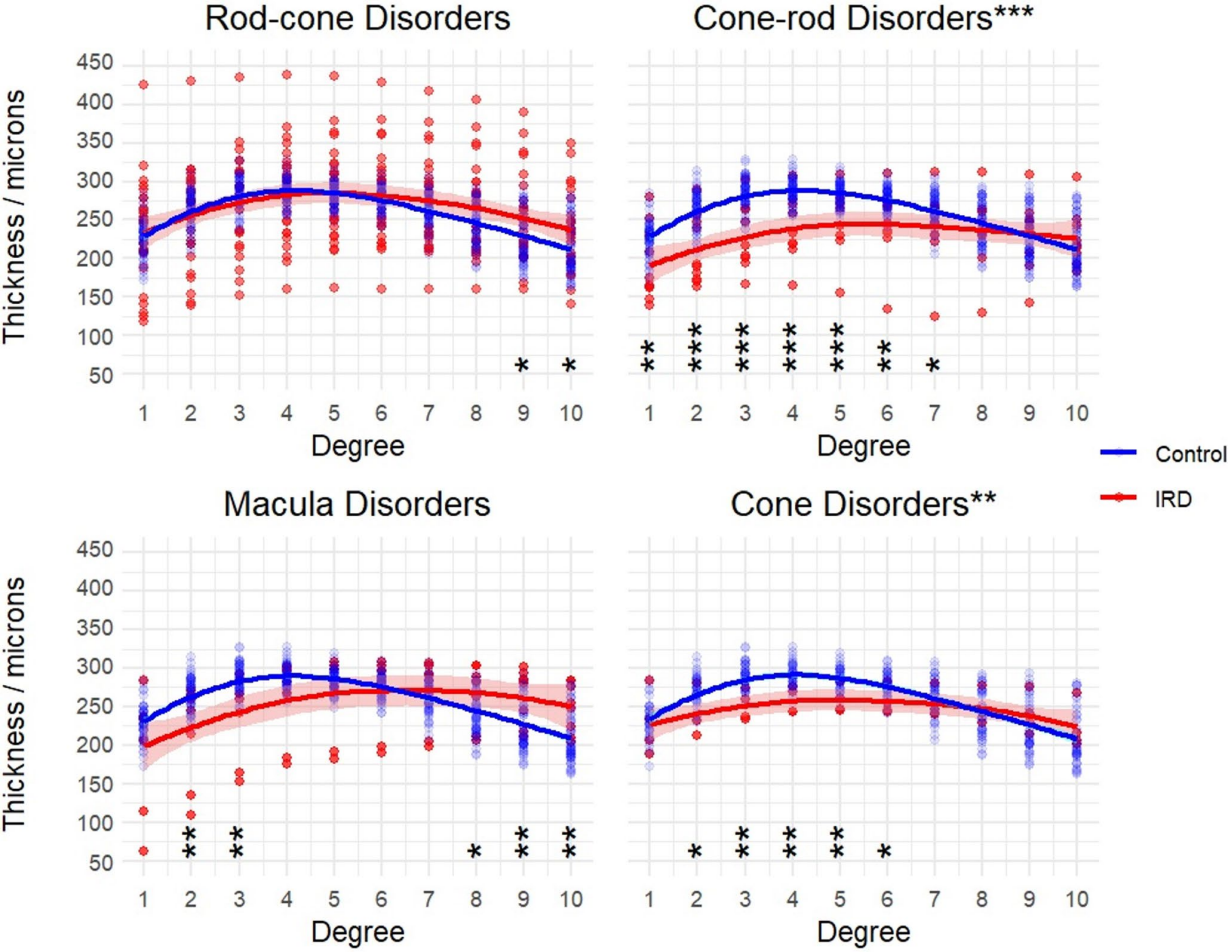
Total thickness was overall significantly thinner in cone and cone-rod IRD groups versus age-matched controls – particularly in the paramacular region – but not in macula or rod-cone groups. Shading indicates standard error. P-values for post-hoc Dunn tests with Bonferroni correction displayed for each degree (* <0.025, **<0.01, ***<0.001). **Descriptive caption**: 4 x-y scatter plots comparing total thickness in microns on the y axis and degree on the x axis, for each IRD group. In each plot the line with error shading indicates the IRD groups, versus the line without error shading, showing the results from the control group

Total thickness was overall significantly reduced in cone and cone-rod IRDs compared to age-matched controls (Fig. 4; cones H(1) = 8.2, *p* = 0.004; cone-rod H(1) = 37.3, *p* < 0.0001), with the greatest difference appearing in the para-macula area. Macula and rod-cone IRDs did not display a significant difference versus healthy volunteers (macula H(1) = 0.11, *p* = 0.74; rod-cone H(1) = 1.3, *p* = 0.25).

### Age and sex

#### Age-matched groups were used to control for the effect of age

Amongst controls, sex was overall observed to impact upon total thickness (H = 6.2, *p* = 0.013) and for inner retinal thickness (H = 9.1, *p* = 0.002), with males tending to have greater thickness at a given degree than females. This effect was more notable at para-macular eccentricities. There was no significant difference between males and females in the photoreceptor complex layers (H = 0.013, *p* = 0.91).

In view of low numbers in IRD groups, sex-matching was not possible. However, the male to female ratio remained in a narrow range across all groups. A further exploration of the impact of age and sex upon retinal thickness is included in the supplementary information online (online resource 2).

## Discussion

We present measurable differences in retinal OCT parameters between a wide array of retinal genetic disorders and age-matched healthy volunteers in a paediatric population. Specifically these changes are demonstrated in both the PRC and IR. Of the 51 IRD participants, only 8 had systemic disorders, with the remainder being primary photoreceptor conditions. This would suggest that changes in the IR are secondary changes as a result of retinal remodelling, as has previously been reported [11, 13]. We therefore show that the retinal remodelling process starts earlier than has previously been demonstrated and can be shown in a paediatric population.

Inner retinal thickening appears to occur more widely across the retina than photoreceptor complex changes. This is surprising and unexpected as it might be expected to be the opposite way round, particularly with early retinal remodelling [14]. These changes may represent retinal remodelling arising as part of an inflammatory response [15].

Studying the paediatric population provides us with a window into a very early stage of the remodelling process, before large scale retinal atrophy has occurred. Relying only upon total thickness as a marker of disease could be misleading, as it could mask several different disease processes.

Our cohort had low numbers of individuals with specific IRDs, and a mixed population of IRDs. This prevented examining the unique early microstructural changes for each type of IRD, and instead we grouped participants more broadly into categories based upon electroretinographic phenotype and general mode of action of genetic variants. There is ongoing debate regarding the correct system of classification for IRDs [16]. As IRDs are individually rare diseases, collectively they form the largest cause of uncorrectable visual impairment in the working age population [2, 3]. Only by grouping the patients together is it possible to have enough numbers to perform any reasonable group level analysis. Although this may remove the nuances between the individual genetic mutations, it allows patterns to be drawn based on the phenotypic presentation.

This study makes an important start at the process of describing early changes in a paediatric population, thereby starting the conversation about how early retinal remodelling occurs. We propose that prospective natural history studies should be widened to include younger participants to ensure that the early disease process is captured, ideally in specific types of IRDs. A further limitation is the potential effect of extreme myopia amongst in some IRD patients on macular thickness [17].

We acknowledge the potential impacts of age and sex upon our results (online resource 2). In particular, we would predict that age of disease onset/disease duration could influence results; this information was not available to us, and longitudinal study would help address this question. This will be more critical than age as an independent risk factor as it will be a determinant of disease progression. Sex has previously not been shown to be a factor affecting retinal layer thickness in children [18], so differences in our study may be secondary to axial length differences as a result of asymmetries from refractive errors that may have been present within the cohort, due to changes in the ocular shape and size causing changes in thickness measurements [19].

Although it would increase the accuracy of our models to include disease severity, due to the wide array of IRDs, classifying severity was complicated as macular severity would be related to visual acuity loss or central atrophy [20]. However, rod-cone dystrophies preserve visual acuity until late in the disease stage, so disease severity is graded on ellipsoid zone or degree of visual field loss. Additionally, as we are dealing with rare diseases, it is very difficult to find enough patients for studies with these diseases. Splitting the groups up further into severity would make the groups so small that no reasonable group-based analysis could be undertaken. Without information on disease severity, the generalisability of our conclusions could be limited; the patterns observed in the results may have arisen due to the particular cohort of children participating in the study. We can also speculate that there may have been some selection bias towards in recruitment, as children with minimal impact from their condition or with minimal signs seen on imaging may have been less likely to have attended follow-up appointments or have follow up appointments ordered. We can surmise that our cohort is likely to be biased towards more severe cases.

Despite these limitations, our data shows that retinal remodelling in inherited retinal diseases is measurable in children, and consequently that such changes are taking place early in the disease process – potentially from as young as 4 years of age. This may have implications for future treatment and monitoring response to treatment.

## Conclusions

Many IRDs have onset early in life. It is therefore important to screen the retina of children carefully using OCT to accurately measure retinal thickness. This may demonstrate signs of retinal remodelling, even in childhood. Paediatric populations must be included in prospective natural history studies for inclusion in future clinical trials.

## Supplementary Information

Below is the link to the electronic supplementary material.

Supplementary File 1 (DOCX 21.1 KB)

Supplementary File 2 (DOCX 254 KB)
