# Supplementary material for: Characterising microstructural retinal changes in children with inherited retinal dystrophies – a retrospective observational cross-sectional study
Source: Graefes Arch Clin Exp Ophthalmol. 2025 Oct 15;263(12):3537–45. doi: 10.1007/s00417-025-06983-7 (PMC12886368; doi:10.1007/s00417-025-06983-7)
Supplement: Supplementary file 1 — (DOCX 21.1 KB) [file 417_2025_6983_MOESM1_ESM.docx]

**Online resource 1**

|  |  | **Mean thickness (SD, range) / microns** | | | | | | | | | |
| --- | --- | --- | --- | --- | --- | --- | --- | --- | --- | --- | --- |
| **Layer** | **Degree** | **1** | **2** | **3** | **4** | **5** | **6** | **7** | **8** | **9** | **10** |
|  | **IRD group** |  |  |  |  |  |  |  |  |  |  |
| **Total thickness** | Control | 226.6 (23.6, 171.6-285) | 264.2 (23.2, 207.7-313.8) | 284.3 (19.6, 237.6-328) | 287.3 (16.3, 255.4-327.7) | 282.9 (14.9, 247.7-318.8) | 276.6 (15.3, 241.1-310.3) | 262.9 (21, 205.7-307.7) | 243 (27.3, 187.5-292.5) | 227.1 (32, 174.3-294.2) | 213.4 (34.1, 163.1-281.2) |
|  | Rod-cone | 234.4 (67.8, 118.2-424.9) | 254.6 (64.1, 139.2-430.2) | 274.6 (62.1, 152.9-435.6) | 284.8 (61.2, 160.3-438.6) | 286.3 (60.7, 161.2-437.4) | 282.2 (59.8, 160.3-428.6) | 275.3 (59, 160.2-417.3) | 266.7 (58.3, 159.7-406.6) | 256.2 (56.8, 159.5-390.1) | 236.3 (50.2, 140.4-349.5) |
|  | Cone-rod | 194.9 (46.3, 139-280.4) | 211.6 (42.3, 162.7-288.9) | 229.2 (39, 167.3-302.9) | 241.1 (38.3, 165.3-307.4) | 246.1 (39.8, 154.7-309) | 246 (43.8, 135.1-310.7) | 243.1 (46.6, 124.1-312.2) | 237.7 (45.2, 129.9-311.4) | 233.8 (43, 142.8-309.2) | 230 (36.6, 182.3-306) |
|  | Macula | 199.8 (69.2, 62.6-283.2) | 218.6 (59.5, 109.4-270.5) | 241.1 (51.3, 153.4-291.3) | 256.8 (48.5, 175.8-302.4) | 264.1 (47.8, 182.7-307.5) | 267.6 (45.5, 190-308.2) | 266.1 (40.9, 199-306.6) | 264.9 (37.2, 205.6-303.5) | 262.2 (33.9, 210.6-302.3) | 247.4 (30.6, 204-284.3) |
|  | Cone | 228.3 (41.8, 189.1-284.5) | 239.1 (28.9, 212.1-280) | 251.4 (19, 234.5-274.4) | 257.8 (16.8, 243.3-275.6) | 258.6 (17.6, 244.4-282.4) | 256.1 (18.3, 242.7-282.6) | 252.5 (18.6, 240.9-280.1) | 247.7 (20.8, 228.9-277.4) | 240.9 (26.3, 214.5-276.4) | 223.1 (30.7, 202-268.3) |
| **Inner retina** | Control | 46.1 (18.1, 20-109.5) | 89.7 (18.5, 49-130.4) | 116.1 (14.9, 79.2-138.5) | 125.1 (10.8, 98.8-145.2) | 126.4 (8.6, 109.9-144) | 125.8 (8.1, 107.3-142) | 120.9 (10.8, 90.2-144.7) | 111.8 (14.1, 79.2-137.9) | 104.4 (16.9, 72.2-140.5) | 98.1 (18.8, 67-140.3) |
|  | Rod-cone | 82.7 (40.8, 33-194.6) | 113 (32.2, 75.7-211.8) | 142.2 (31.8, 92.7-243.2) | 159.5 (34, 104.5-266) | 167.4 (36, 107.8-277.5) | 168.8 (36.8, 108.2-279.3) | 167 (37.1, 108.9-277) | 163.4 (36.7, 109.3-269.7) | 158.3 (35.8, 104.7-262.2) | 146.5 (31.1, 88.3-215.6) |
|  | Cone-rod | 86.8 (36, 27.7-152.5) | 109.1 (24.7, 61.5-152.1) | 129.7 (25.5, 87-185.5) | 142 (26.1, 105.7-200.7) | 147.3 (27.3, 101.6-206.5) | 147.7 (30.7, 87.8-210.2) | 145.6 (31.9, 80.1-213) | 142.2 (31, 84.5-213.7) | 140.2 (30, 94.6-213.3) | 136.9 (30.6, 99.4-213) |
|  | Macula | 76.1 (42.9, 23.6-143.7) | 102.8 (28.1, 62.4-150.1) | 126.2 (17.4, 100.7-155.9) | 141.1 (14.3, 119.1-162.9) | 147.7 (14, 124-166.6) | 149.3 (14.5, 124.1-165.7) | 147.1 (14, 122.4-163) | 145.6 (14, 121.1-162.8) | 144.2 (14, 121.1-162.8) | 135.7 (13.5, 119.4-154.2) |
|  | Cone | 94.3 (77.4, 7.1-284.5) | 97.3 (78.7, 6.7-280) | 101 (82.3, 7.3-274.4) | 102.8 (84.7, 7.6-275.6) | 102.9 (85, 8.3-282.4) | 101.8 (84.2, 8.5-282.6) | 100.3 (83, 9-280.1) | 98.3 (81.5, 9.5-277.4) | 95.6 (79.5, 9.7-276.4) | 88.7 (73.9, 9.1-268.3) |
| **Photoreceptor complex** | Control | 123.9 (12.8, 75.1-150) | 120.8 (10.6, 85.6-141.2) | 116.4 (9.6, 87.6-136) | 111.6 (9, 84.6-129.9) | 106.7 (8.7, 80.4-125) | 102 (8.8, 76.5-122.6) | 95.5 (10.3, 71.5-120) | 87.8 (11.9, 62.9-117.4) | 81.8 (12.7, 58.2-114.7) | 76.4 (13, 52.6-108.8) |
|  | Rod-cone | 143.8 (51.8, 55.9-246.6) | 132.9 (48.2, 50.5-234.9) | 123 (45.8, 48.1-229.8) | 115.5 (45.1, 45.2-228.4) | 108.9 (44.4, 42.8-224.1) | 103 (44.4, 40.3-219.5) | 97.7 (44.3, 37.3-213.9) | 92.6 (43.9, 35.5-207.2) | 87.3 (42.9, 33.8-198.2) | 80 (39.4, 30.2-177.9) |
|  | Cone-rod | 96.8 (34.1, 40.9-148.5) | 90.8 (30.1, 43.5-123.2) | 87.6 (26.7, 44.3-117.9) | 87 (23.6, 44.7-116.8) | 86.7 (22.7, 42.3-114.3) | 86 (23.6, 36.2-112.4) | 85 (24.5, 32.7-111.4) | 83.2 (23.7, 34.3-106.3) | 81.3 (22.7, 36.9-104.1) | 81.2 (18.5, 36-101.4) |
|  | Macula | 110.6 (54.6, 28.8-171.4) | 102 (46.5, 36.7-155.6) | 101 (41.4, 39.6-148.4) | 101.6 (38.8, 39.6-143.6) | 102.1 (37.3, 41.4-139.3) | 104 (33.9, 46.2-137.2) | 104.6 (29.6, 52.9-134.6) | 104.9 (25.7, 60.7-132.8) | 103.9 (22.6, 66.6-127.3) | 98.2 (20.2, 64.7-119.2) |
|  | Cone | 109 (25.8, 85.8-140.4) | 105.8 (22.1, 83.5-134.4) | 102.9 (19.5, 82.4-128.9) | 101.2 (18.4, 81.7-125.8) | 100 (18.1, 80.2-123.8) | 98.5 (17.8, 79-121.9) | 96.6 (17.8, 77.2-120.2) | 94.3 (19, 72.7-118.8) | 91.6 (20.2, 68.9-117.8) | 85.8 (20, 66.3-113.6) |

Control *n* = 64, Rod-cone *n* = 27, Cone rod *n* = 11, Macula *n* =9, Cone *n* = 4

**Online resource table 1.** Summary thickness data in different layers for each group. **Descriptive caption:** A table showing thickness measurements, with mean and standard deviation values, measured in microns, across degrees of measurement, for each IRD group, in each layer grouping – total thickness, inner retinal and photoreceptor complex thicknesses
