# Supplementary material for: Characterising microstructural retinal changes in children with inherited retinal dystrophies – a retrospective observational cross-sectional study
Source: Graefes Arch Clin Exp Ophthalmol. 2025 Oct 15;263(12):3537–45. doi: 10.1007/s00417-025-06983-7 (PMC12886368; doi:10.1007/s00417-025-06983-7)
Supplement: Supplementary file 2 — (DOCX 254 KB) [file 417_2025_6983_MOESM2_ESM.docx]

**Online resource 2**

**Impact of sex on retinal thickness**

In control groups, statistically significant differences between male and female participant’s inner retinal (H=9.1, p=0.002) and total thickness (H=6.2, p=0.013) were seen, but not in photoreceptor complex thickness (H=0.013, p=0.91)), when comparing across all degrees.

Online resource 2 Figure 1 illustrates these differences. For inner retinal and total thickness measurements, male healthy controls tended to have greater thickness at a given degree than females, in the para-macular region, but not in the periphery. As discussed in the main text, this finding differs from previous literature. Despite the statistical difference between male and female group thickness, the actual differences in thickness values are generally small.

**Online resource 2 Figure 1**


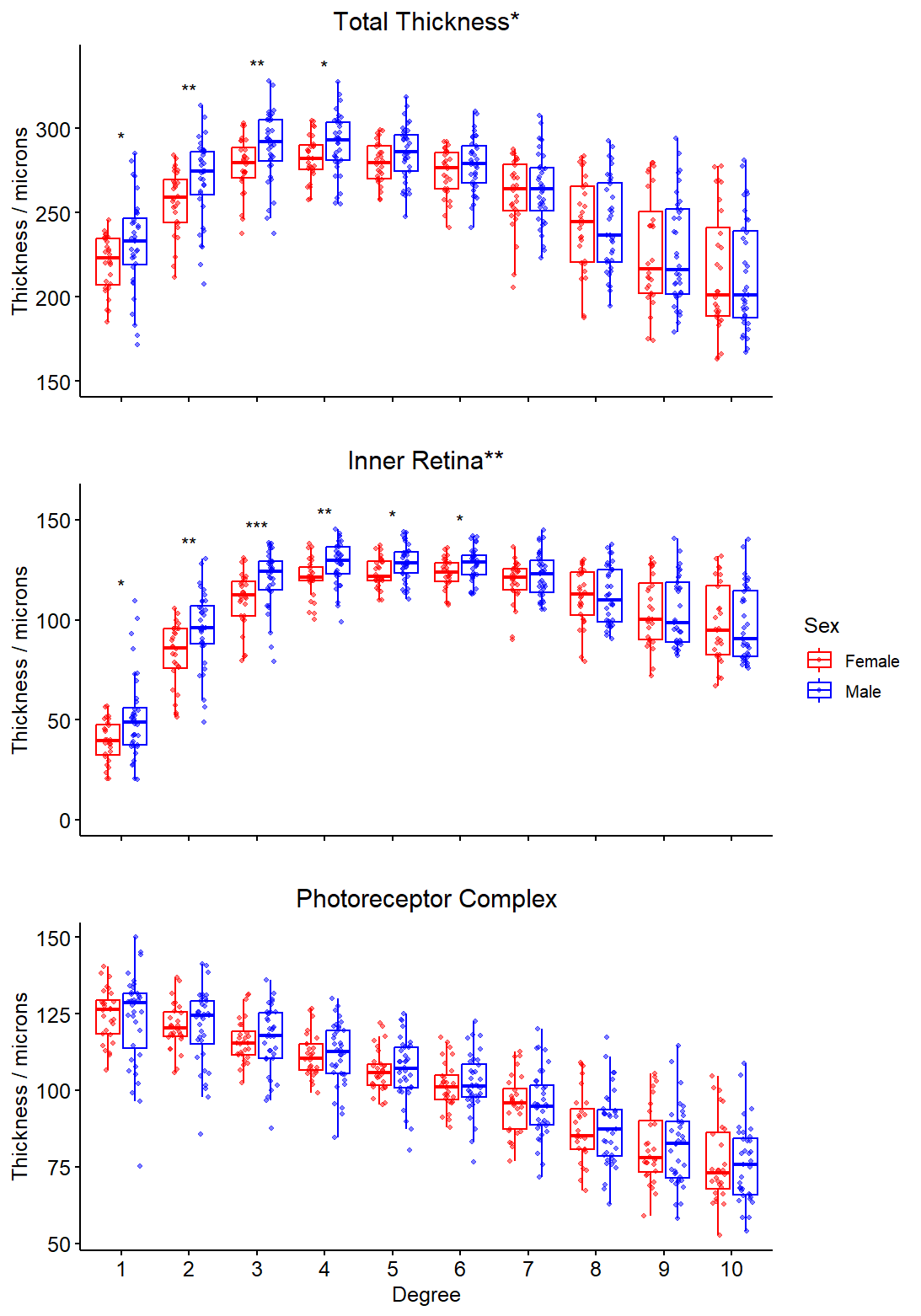


**Online resource 2 Fig. 1** Jittered box plots showing difference between thickness at different eccentricities between female and male control participants. P-values for post-hoc Dunn tests with Bonferroni correction displayed for each degree (* <0.025, **<0.01, ***<0.001). **Descriptive caption:** three jittered box plots, showing thickness across degree in different layer divisions, comparing between females – on the left side of pair for each degree – the and males – on the right side of the pair for each degree

**Impact of age on retinal thickness**

There was no clear correlation between control participant age and thickness for many degrees eccentricity in different layers, but some combinations of degree and layer appeared to show a monotonic relationship between age and thickness.

These trends were further investigated using generalised additive models. The assumptions of the models were not completely satisfied for every combination of layer and degree. We summarise the findings of our models below.

**Online resource 2 Figure 2**


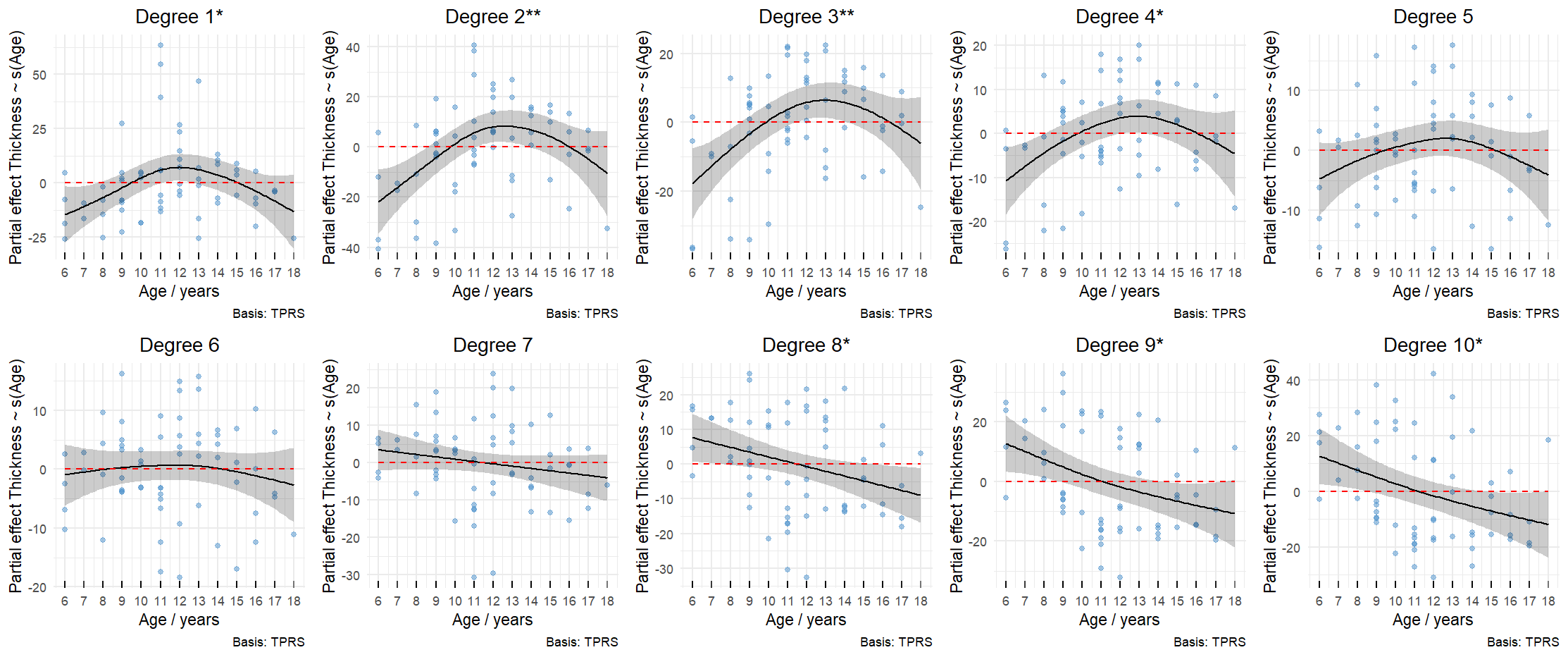
**Online resource 2 Fig. 2** Generalised additive models for inner retinal layers. Individual model created for each degree. Collectively the models suggest an impact of age upon thickness which is more pronounced in the para-macular region and in the periphery. **Descriptive caption:** Two rows of 5 scatter plots, comparing age in years on the x axis and the modelled partial effect of thickness on the y-axis. The dots show data points. The solid line is the line of best fit. The dotted line indicates the reference line where y=0


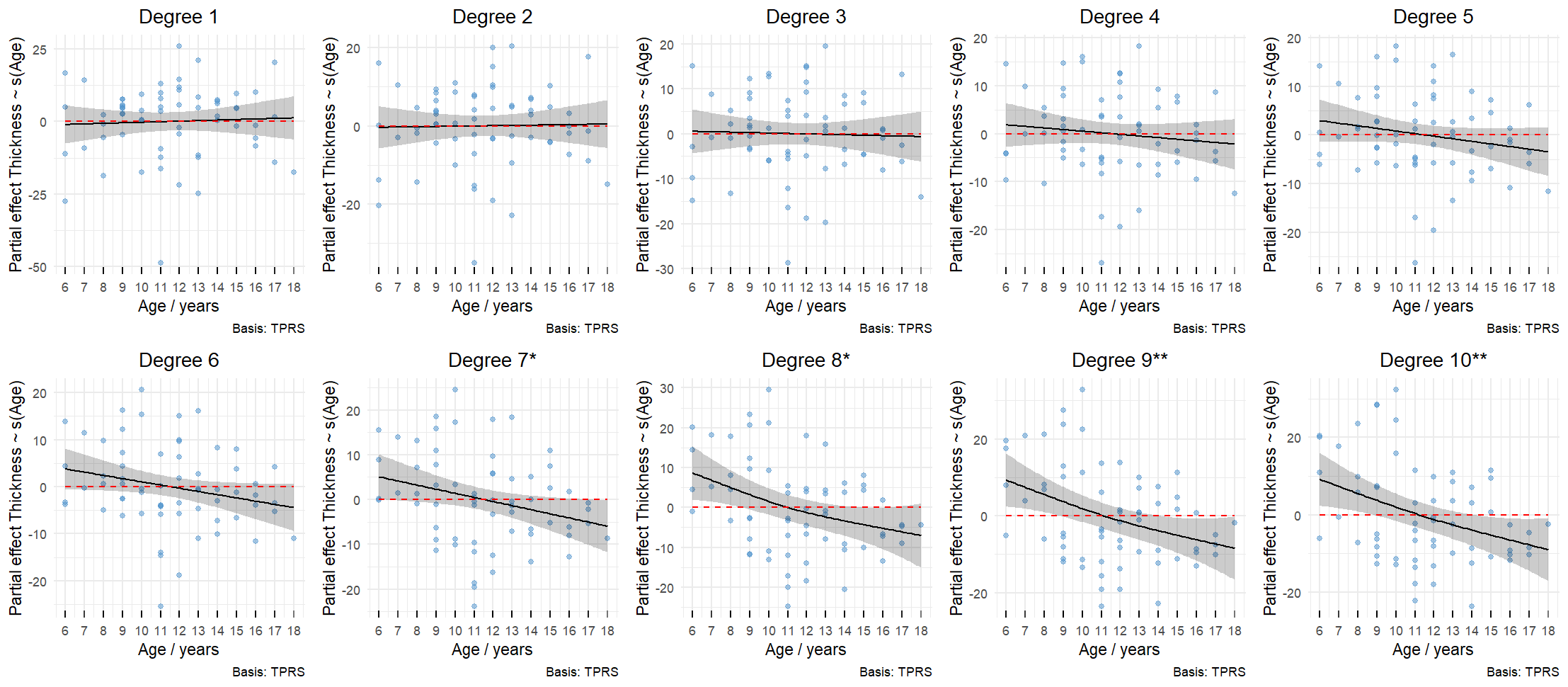
**Online resource 2 Figure 3**

**Online resource 2 Fig. 3** Generalised additive models for photoreceptor complex layers. Individual model created for each degree. The models suggest an impact of age upon thickness in the peripheral retina, but not in the para-macular region. **Descriptive caption:** Two rows of 5 scatter plots, comparing age in years on the x axis and the modelled partial effect of thickness on the y-axis. The dots show data points. The solid line is the line of best fit. The dotted line indicates the reference line where y=0

**Online resource 2 Figure**
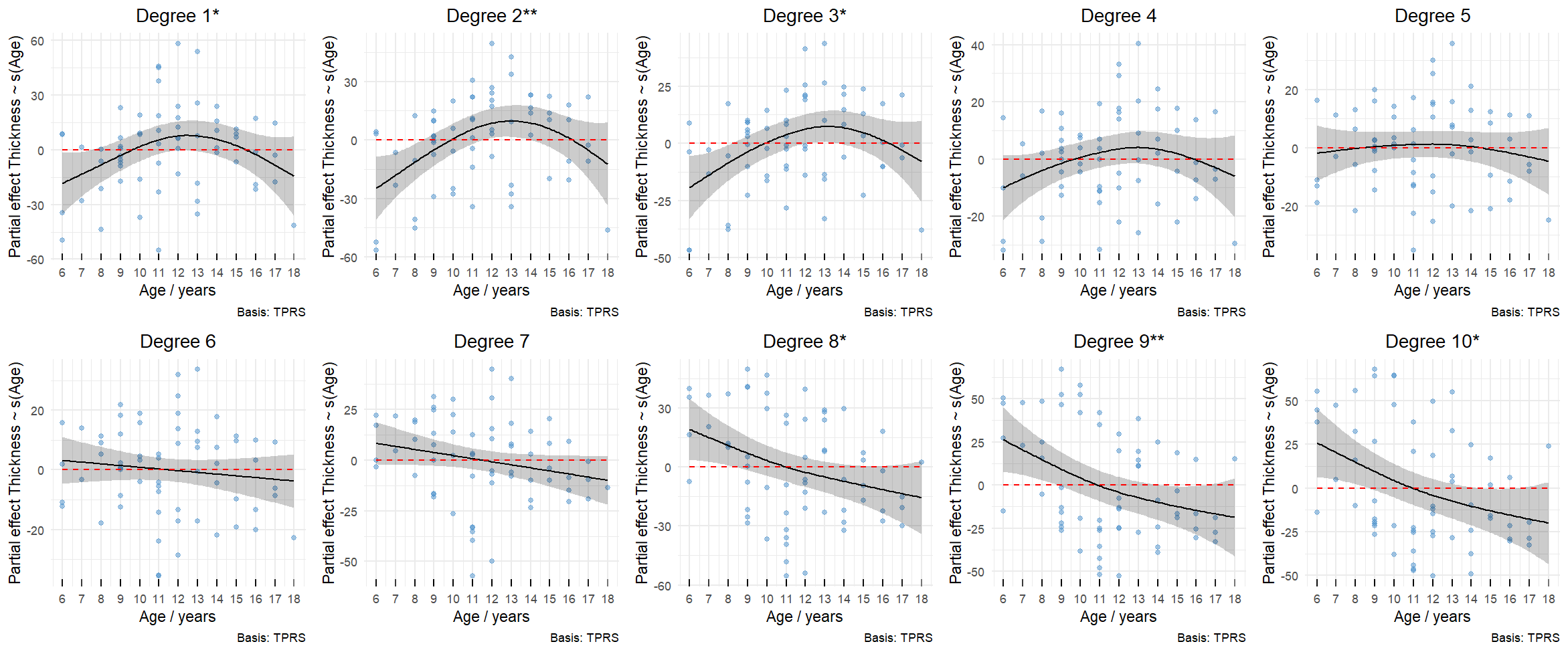
**4**

**Online resource 2 Fig. 4** Generalised additive models for total thickness. Individual model created for each degree. The models suggest an impact of age upon thickness in the para-macula and peripheral retina. **Descriptive caption:** Two rows of 5 scatter plots, comparing age in years on the x axis and the modelled partial effect of thickness on the y-axis. The dots show data points. The solid line is the line of best fit. The dotted line indicates the reference line where y=0
